# The bacterial effector protein YopM reduces rheumatoid arthritis (RA) outcome by inhibiting inflammation and bone destruction

**DOI:** 10.1186/ar3609

**Published:** 2012-02-09

**Authors:** J Bertrand, C Rueter, C Cromme, J Scharnert, A Schmidt, T Pap

**Affiliations:** 1 Experimental Medicine and Rheumatology, William Harvey Research Institute, London, UK; 2 Institute of Infectiology, ZMBE, Muenster, Germany; 3 Institute of experimental musculoskeletal medicine, University hospital Muenster, Muenster, Germany

## 

Osteoclasts mediate the degradation of bone during RA and are derived from macrophages. The yersinia outer protein M (YopM) is an effector protein of *Yersinia *species that is able to enter host cells by membrane penetration. In the cell YopM mediates down-regulation of inflammatory responses. We investigated whether YopM has the potential to act as a "selfdelivering" immune therapeutic agent by reducing the inflammation and joint destruction linked to RA.

Using confocal laser scanning we analysed the penetration of recombinant YopM into bone marrow macrophages (BMMs). Furthermore we studied the effects of YopM on osteoclastogenesis using *in vitro *osteoclast formation assay. To unravel the signaling pathways of YopM, we tested for phosphorylation of MAP-kinases (ERK, AKT and p-38) and activation of NF-KB signaling by Western Blot analysis. With respect to a potential *in vivo *application of YopM, we injected YopM intra articular and intravenous in mice and monitored the distribution by fluorescence reflection imaging (FRI). We treated hTNFtg mice, as animal model for RA, with YopM and recorded clinical parameters (weight, grip strength and paw swelling). Finally we analysed the destruction of bone and cartilage histologically compared to untreated hTNFtg mice and wildtype mice.

As seen in confocal scanning microscopy, YopM penetrated the cell membrane of BMMs and accumulated near the nucleus. Studying the signaling pathways affected by YopM, we found that YopM reduced the TNFa induced activation of NF-kB via reducing the phosphorylation of IkBa. TNFa mediated phosphorylation of MAP kinases were not altered by YopM. Most interestingly, we found a strong reduction of osteoclast formation by YopM. Incubation of BMMs with YopM led to a 90% reduction in osteoclasts precursors and osteoclasts. YopM-Cy5 injected into the hind paws of hTNFtg mice was detectable in the joint without a systemic distribution for 48 hours and elimination mediated through renal clearance.

Analysing the clinical parameters of RA in hTNFtg mice, we observed a delay of onset of paw swelling in mice treated with YopM. At histological analysis of the hind paws, we found reduced bone destruction and decreased osteoclast formation, as well as less inflammation in YopM treated hTNFtg mice in comparison to untreated hTNFtg mice.

These results suggest that YopM has the potential to reduce inflammation and bone destruction in vivo. For this reason YopM may constitute a novel therapeutic agent for the treatment of RA.

